# Lipedema in Men: A Retrospective Case Series of Five Patients From a Brazilian Referral Center

**DOI:** 10.7759/cureus.87332

**Published:** 2025-07-05

**Authors:** Alexandre C Amato, Juliana S Amato, Daniel Benitti, Keller D Santos

**Affiliations:** 1 Vascular Surgery, Amato Instituto de Medicina Avançada, São Paulo, BRA; 2 Gynecology, Amato Instituto de Medicina Avançada, São Paulo, BRA; 3 Vascular and Endovascular Surgery, Medical Valens Center, São Paulo, BRA; 4 Vascular Surgery, Hospital Amato, São Paulo, BRA

**Keywords:** adipose tissue diseases, bioimpedance analysis, case series, fat disorders, gluten-free diet, hla-dq2, hla-dq8, lipedema, male lipedema

## Abstract

Lipedema is a chronic adipose tissue disorder traditionally considered to affect almost exclusively women, with recent estimates suggesting approximately 0.2% prevalence in men worldwide; the condition remains underdiagnosed in males due to limited awareness and scarce literature. This retrospective case series from the Amato Institute of Advanced Medicine in São Paulo, Brazil, describes clinical characteristics, diagnostic findings, and treatment outcomes of five male patients diagnosed with lipedema between January 2022 and December 2024. The patients, aged 31-58 years (mean, 42.6 ± 9.7), with a BMI of 29-42.4 kg/m², all presented bilateral, symmetrical lower extremity fat accumulation, sparing the feet, with endocrine comorbidities present in 80% of cases and one participant testing positive for HLA-DQ2/DQ8. Diagnosis was based on clinical criteria requiring bilateral symmetrical fat accumulation, disproportionate fat distribution, negative Stemmer’s sign, sparing of feet, and at least two minor criteria. Conservative management, including dietary interventions over four to eight weeks, resulted in mean weight reduction of 7.0 ± 2.2 kg and lower limb volume reduction of 2.5 ± 1.1 L. These findings demonstrate that lipedema occurs in men with classical phenotypic features, and the presence of HLA-DQ2/DQ8 markers in some cases suggests potential autoimmune components and opportunities for targeted dietary interventions. Conservative management yields significant short-term improvements, warranting larger prospective studies to establish prevalence, investigate HLA associations, and optimize management strategies for male lipedema.

## Introduction

Lipedema is a chronic, progressive disorder of adipose tissue characterized by bilateral, symmetrical accumulation of subcutaneous fat predominantly in the lower extremities, typically sparing the feet [1]. First described by Allen and Hines in 1940 at the Mayo Clinic, this condition has been historically considered to affect almost exclusively women, with onset typically occurring during periods of hormonal change such as puberty, pregnancy, or menopause [1,2].

Lipedema shows familial clustering and is thought to follow an autosomal-dominant inheritance pattern with sex-influenced expression [3]. The genetic basis supports the observed familial predisposition, though the mechanisms underlying male manifestation remain poorly understood.

Recent epidemiological data suggest that the prevalence of lipedema varies considerably across populations. Current estimates indicate that up to 18% of women worldwide may be affected, while approximately 0.2% of men have the condition [4,5]. In Brazilian women specifically, prevalence has been reported at 12.3% [6]. These variations likely reflect differences in diagnostic criteria, population characteristics, and awareness among healthcare providers.

The underdiagnosis of lipedema, particularly in men, stems from multiple factors. The condition remains absent from many medical curricula and specialty training programs [7]. Furthermore, lipedema is frequently misdiagnosed as simple obesity, lymphedema, or chronic venous insufficiency [8]. This diagnostic challenge is compounded in male patients, where the index of suspicion is particularly low.

Despite this relatively common disease, there are few physicians who are aware of it. The diagnosis of lipedema is clinical and mainly relies on history and clinical evaluation [9]. This lack of awareness contributes significantly to delayed diagnosis and inappropriate management.

Contrary to earlier beliefs, cases of lipedema in men have been documented since 2003. Chen et al. reported the first English-language case of “painful fat syndrome” in a male patient in 2004 [10]. Since then, scattered case reports have emerged, suggesting that male lipedema may be more common than previously recognized [11-13].

The World Health Organization’s recognition of lipedema in the 11th revision of the International Classification of Diseases (ICD-11) in 2019, with codes EF02.2 and BD93.1Y, represents a significant milestone [14]. The recent publication of S2k clinical practice guidelines in 2024 provides standardized diagnostic criteria [15], while a proposed research case definition framework aims to harmonize clinical studies [16].

This case series presents five male patients with lipedema from a Brazilian referral center, highlighting clinical characteristics, diagnostic findings, and treatment outcomes to increase awareness of this underrecognized condition in men.

## Case presentation

We conducted a retrospective review of male patients diagnosed with lipedema at the Amato Institute of Advanced Medicine, São Paulo, Brazil, between January 2022 and December 2024. Diagnosis was established using standardized clinical criteria requiring bilateral symmetrical fat accumulation, disproportionate fat distribution, negative Stemmer’s sign, sparing of feet, and at least two minor criteria (pain, easy bruising, orthostatic edema, family history, or hormonal onset). Volume reduction was measured using multifrequency bioimpedance analysis. All patients provided informed consent for data use.

**Table 1:** Clinical stages of lipedema (including secondary lymphedema) This table summarizes the four stages of lipedema, detailing characteristic skin changes, adipose tissue alterations, and key clinical features. Stage IV denotes combined lipedema and secondary lymphedema, with both adipose and lymphatic involvement.

Stage	Skin appearance	Adipose tissue changes	Clinical features
I	Smooth surface	Homogeneous subcutaneous fat thickening; fine nodularity on palpation	Even limb contour; tenderness to pressure; easy bruising
II	“Mattress” or “orange-peel” appearance	Larger nodules; irregular fat lobulation	Skin indentations and depressions; moderate pain; frequent bruising
III	Coarse, uneven surface with pronounced lobules	Large, pendulous fat lobes (“overhanging”); marked volume increase	Significant deformity; functional limitation; chronic discomfort
IV	Features of lipedema plus secondary lymphedema	Lipedema fat changes combined with lymphatic fluid accumulation; fibrosis	Persistent non-pitting and pitting edema; positive Stemmer’s sign; skin thickening; recurrent infections (e.g., cellulitis)

Case 1

A 58-year-old male with severe obesity (BMI, 42.4 kg/m²) presented with persistent medial thigh fat deposits following bariatric surgery. Despite weight reduction from 240 to 190 kg post-surgery, he maintained disproportionate lower extremity fat accumulation. He reported burning sensations in his feet during exercise and had developed ochre dermatitis on both lower legs.

**Figure 1:**
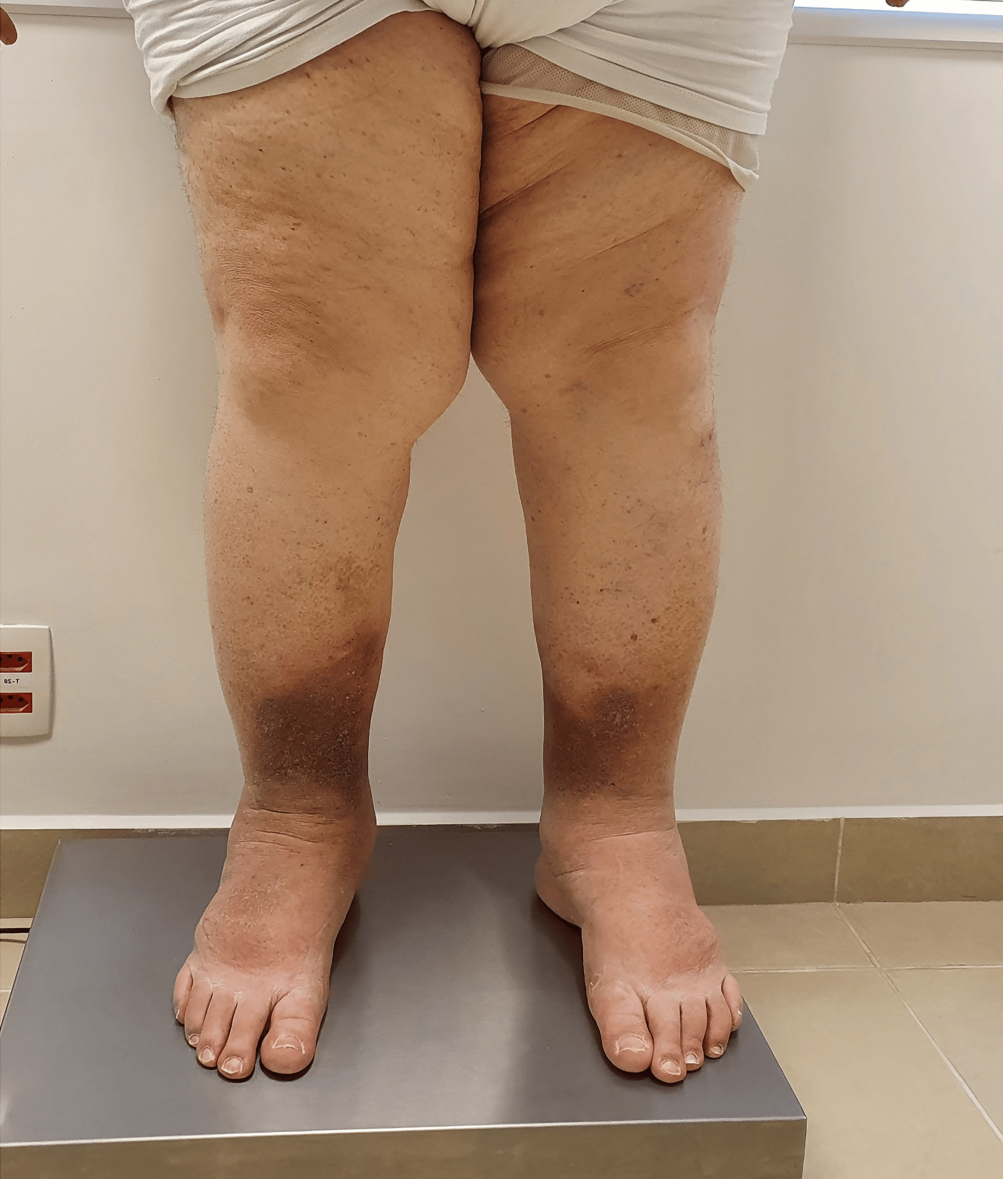
Case 1: anterior view of the lower limbs of a 58-year-old male patient with stage III lipedema Note the bilateral, symmetrical subcutaneous fat deposition in the proximal and medial thighs and legs, with a sharp demarcation above the ankles and sparing of the feet. There is hyperpigmentation consistent with ochre dermatitis around the ankles and mild edema of the right foot. Photograph taken at baseline, prior to initiation of conservative management.

Physical examination revealed bilateral, symmetrical fat deposits predominantly in the medial thighs with clear demarcation at the ankles (Figure 1). Stemmer’s sign was negative. The patient experienced tenderness on palpation of the affected areas.

Multifrequency bioimpedance analysis (Tanita BC-601, Tanita Corporation, Tokyo, Japan) demonstrated disproportionate fat distribution with a higher percentage in the lower limbs compared to the upper body. The patient was classified as stage III lipedema according to the Schingale classification due to the presence of overhanging fat deposits.

Conservative management was initiated, including dietary counseling with endocrinology referral and a structured exercise program emphasizing low-impact activities. After eight weeks of treatment, the patient achieved 5 kg weight loss and 1.5 L volume reduction in the lower limbs as measured by bioimpedance analysis.

Case 2

A 31-year-old male with type 1 diabetes since seven years old presented with progressive leg fat accumulation over five years. His mother had a similar body habitus, suggesting familial predisposition. Despite maintaining good glycemic control with insulin therapy and engaging in regular weight training three times weekly, he noted persistent lower extremity disproportion.

**Figure 2:**
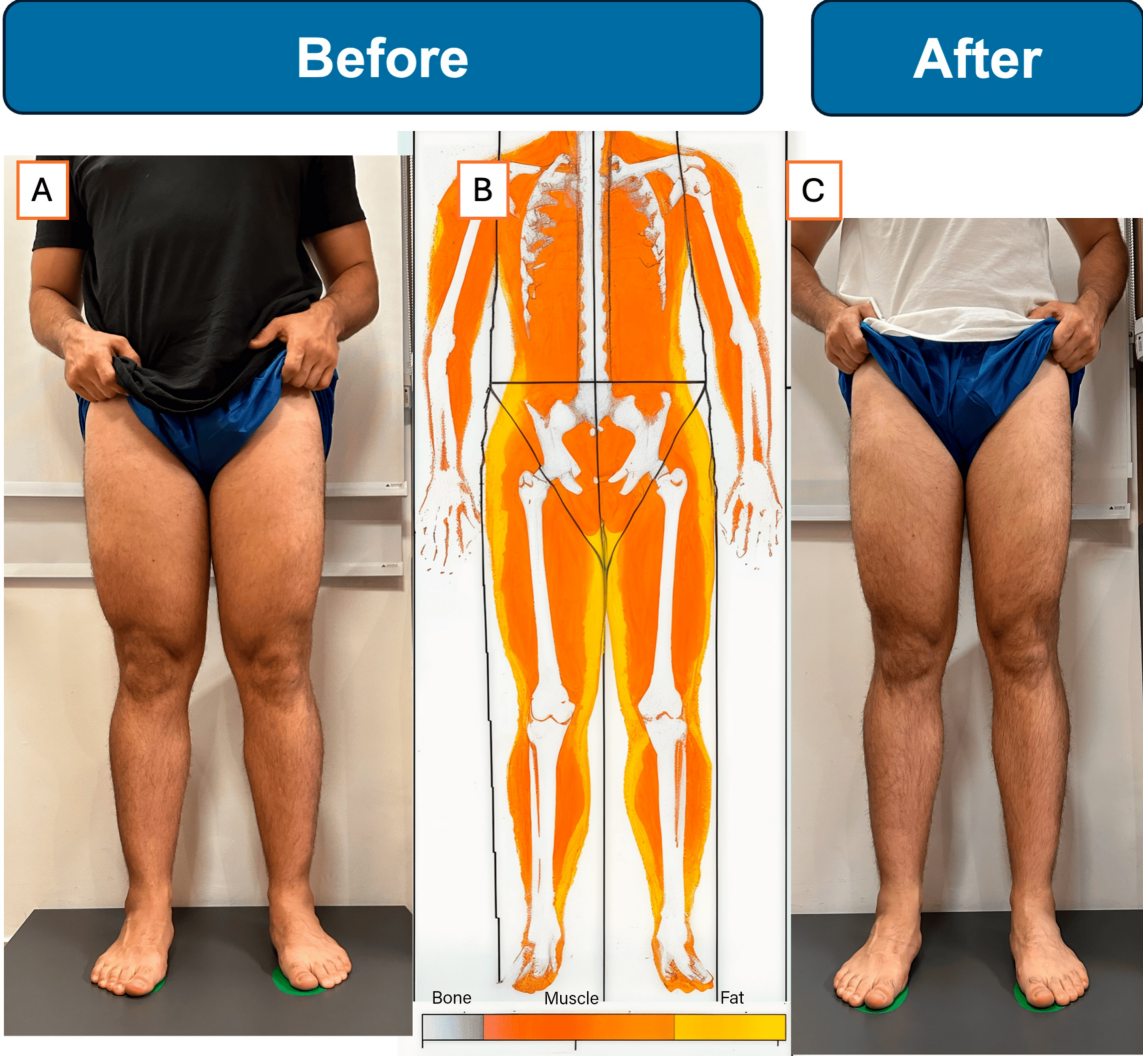
Case 2: before and after images with segmental bioimpedance fat distribution of a 31-year-old male with lipedema (A) Baseline anterior view showing bilateral, symmetrical adipose tissue enlargement predominantly in the thighs and calves, with clear sparing of the feet. (B) Color-coded segmental bioimpedance analysis map at baseline: orange and red areas indicate elevated fat percentage in the lower limbs compared to the trunk. (C) Anterior view after eight weeks of conservative management (ketogenic diet, structured exercise, and no surgical intervention) demonstrating visible reduction in thigh circumference and improved lower-limb contour.

Clinical examination showed a BMI of 29 kg/m² with bilateral, symmetrical fat accumulation in thighs and calves, sparing the feet. He reported mild pain and easy bruising in the affected areas. Bioimpedance analysis revealed overall body fat of 29.9% but significantly higher percentages in the legs (left, 36.3%; right, 34.2%) (Figure 2).

Laboratory evaluation included HLA typing, which revealed DQ2+ and DQ8+ markers. Venous Doppler ultrasound of the lower extremities showed no evidence of reflux or thrombosis. The finding of HLA-DQ2 and DQ8 positivity is particularly relevant given recent evidence suggesting a higher prevalence of these markers in lipedema patients and potential benefits of gluten-free dietary interventions.

The patient was diagnosed with stage II lipedema and managed conservatively without surgical intervention. Treatment included dietary optimization with consideration of gluten-free approaches, given his HLA positivity, and continuation of his exercise regimen. After eight weeks, he achieved 5 kg weight loss and 1.5 L reduction in lower limb volume, with reported improvement in pain symptoms.

Case 3

A 44-year-old male (BMI, 30.2 kg/m²) presented with lifelong thick legs that remained disproportionate despite weight fluctuations. Family history was significant for a similar presentation in his mother and two sisters. He avoided wearing shorts due to embarrassment about his leg appearance and found compression stockings uncomfortable.

**Figure 3:**
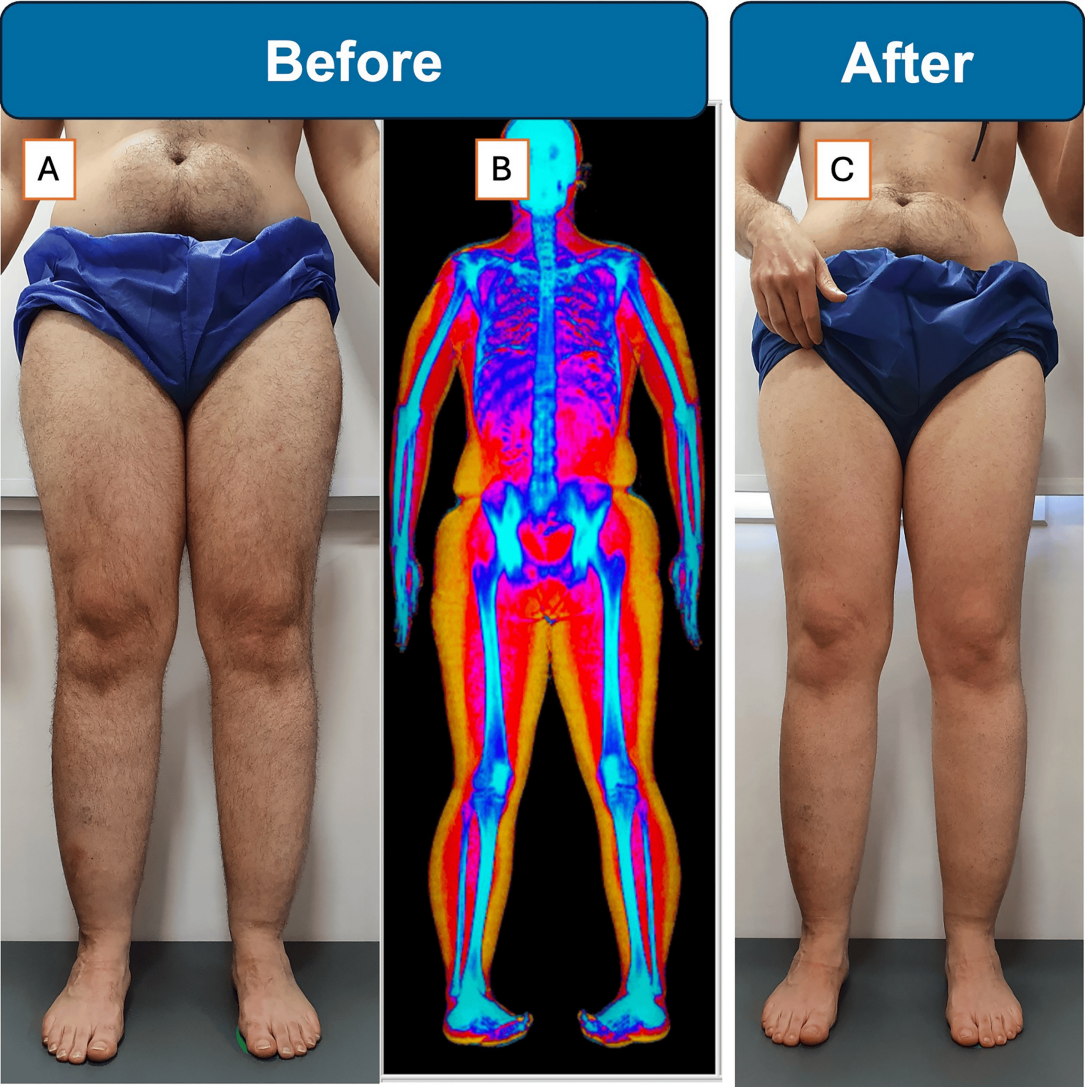
Case 3: before and after images with DXA fat distribution of a 44-year-old male with lipedema (A) Baseline anterior photograph demonstrating pronounced, symmetrical fat accumulation in the thighs and calves with sparing of the feet. (B) Dual-energy X-ray absorptiometry (DXA) map at baseline: warm colors indicate higher fat concentration in the lower extremities relative to the trunk. (C) Anterior photograph after four weeks of combined ketogenic diet, semaglutide, and lisdexamfetamine therapy, showing marked reduction in thigh girth and more uniform limb contour.

Physical examination confirmed stage II lipedema with fat deposits concentrated in thighs and shins, negative Stemmer’s sign, and sharp demarcation at ankles (Figure 3). No tenderness or signs of lymphatic dysfunction were present.

The patient was already on Ozempic (semaglutide) for weight management and Vyvanse (lisdexamfetamine) for attention deficit disorder, reporting mild side effects including palpitations and morning diaphoresis. He also experienced chronic stress and gastroesophageal reflux.

A ketogenic diet (<20 g carbohydrates daily) was initiated alongside continuation of his medications. Hormonal evaluation was recommended. After four weeks of conservative management, he achieved remarkable results with 10 kg weight reduction and 4 L volume decrease in the lower limbs, representing the best response in our series.

Case 4

A 36-year-old male with severe obesity (BMI, 42.4 kg/m²) and previous gastric band surgery presented with lower body fat predominance and persistent right foot swelling following trauma years prior. He had reduced his weight from 155 to 134.3 kg through diet and exercise, but struggled with high stress levels from managing a restaurant chain.

Bioimpedance analysis revealed visceral fat level of 22, total body fat percentage of 39.8%, and metabolic age of 90 years. Fat distribution showed clear lower body predominance. Laboratory findings included elevated ferritin (342 ng/mL), hyperinsulinemia (28 μU/mL), and vitamin D deficiency (18 ng/mL).

**Figure 4:**
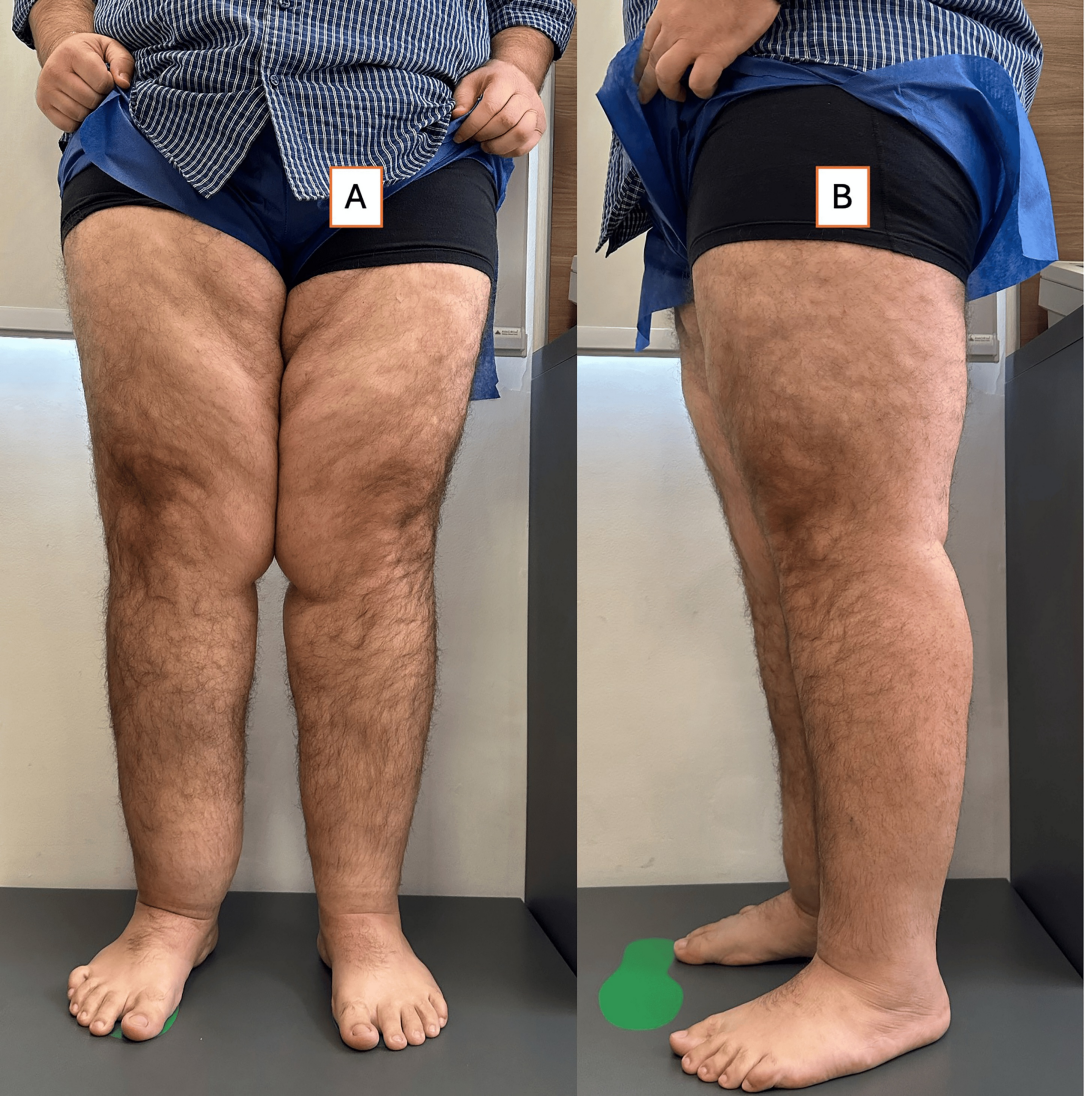
Case 4: before and after anterior and lateral views of a 36-year-old male with stage III lipedema (A) Baseline anterior and lateral photographs showing pronounced, bilateral adipose tissue accumulation in the thighs and calves with sparing of the feet; note the heavier medial thigh pannus and mild right-sided foot swelling. (B) Corresponding anterior and lateral images after six weeks of combined semaglutide, lisdexamfetamine, calorie-restricted diet, and exercise therapy, demonstrating visible reduction in thigh circumference, improved limb contour, and resolution of right foot edema.

Physical examination confirmed stage III lipedema with significant fat deposits and mild lymphedema of the right foot (Figure 4). The patient was treated with Ozempic, and Vyvanse was added for appetite control. He received comprehensive dietary counseling with a structured exercise program. After six weeks, he achieved 7.5 kg weight loss and 2.5 L lower limb volume reduction.

Case 5

A 44-year-old male (BMI, 35 kg/m²) with endocrine dysfunction presented describing his legs as “wide,” a characteristic also present in his sister, who maintained large legs despite bariatric surgery. He developed hypothyroidism following viral thyrotoxicosis and was managed with levothyroxine 50 mcg daily and bupropion for mood.

**Figure 5:**
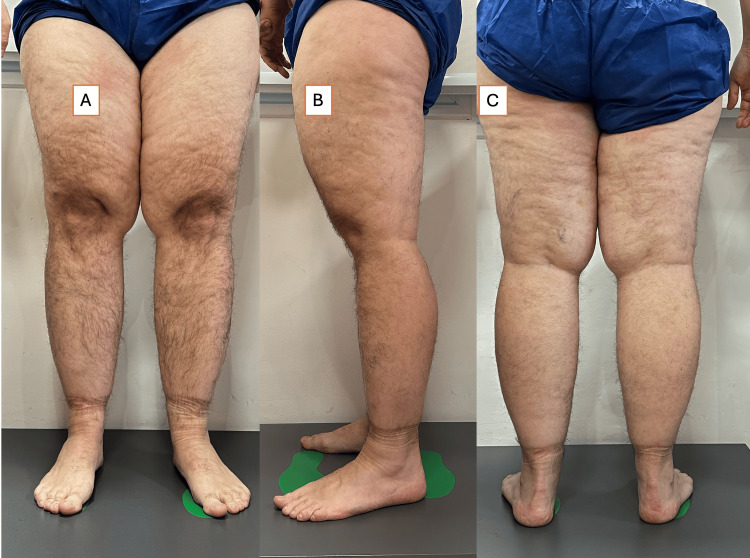
Case 5: baseline anterior, lateral, and posterior views of a 44-year-old male with stage II lipedema (A) Anterior, (B) lateral, and (C) posterior photographs demonstrate bilateral, symmetrical subcutaneous fat accumulation predominating in the thighs and buttocks, with sharp demarcation above the ankles and sparing of the feet. Note the “wide legs” phenotype and relative preservation of lower-leg contour despite overall adipose excess. This patient had post-viral hypothyroidism on levothyroxine and a positive family history (sister), and these images were captured prior to initiation of the ketogenic diet and endocrine follow-up.

Physical examination revealed stage II lipedema with bilateral fat accumulation in the lower extremities and notable capillary fragility (Figure 5). Dual-energy X-ray absorptiometry (DXA) scan confirmed 38.5% total body fat with disproportionate distribution in the lower limbs. Adjusted BMI calculations and fat ratios supported the lipedema diagnosis.

The patient was prescribed a ketogenic, gluten-free diet with regular endocrine follow-up. After six weeks of conservative management, he achieved 7 kg weight loss and 3 L reduction in lower limb volume.

Summary of outcomes

All five patients demonstrated a positive response to conservative management over four to eight weeks with a mean follow-up of 6.2 ± 1.8 weeks. The cohort achieved a mean weight reduction of 7.0 ± 2.2 kg (range, 5-10 kg) and a mean lower limb volume reduction of 2.5 ± 1.1 L (range, 1.5-4.0 L). Notably, all patients with baseline pain reported symptom improvement, and no adverse events related to treatment were observed during the study period. These outcomes suggest that conservative management approaches can yield significant short-term benefits in male lipedema patients.

## Discussion

This case series presents five male patients with clinical features consistent with lipedema, contributing to the limited but growing literature on this condition in men. Our findings align with recent reports suggesting male lipedema may be more prevalent than historically recognized, with current estimates of approximately 0.2% prevalence [4,5].

The clinical phenotype in our male patients closely resembled classical female presentations, with bilateral, symmetrical lower extremity fat accumulation and characteristic sparing of the feet. This finding supports other recent case reports [11-13,17] and suggests that core diagnostic criteria remain consistent across genders. The S2k 2024 guidelines [15], though primarily developed from female populations, proved applicable to our male cohort.

Lipedema is frequently misdiagnosed or confused with primary lymphedema, obesity, and chronic venous insufficiency [18]. Key differential diagnostic features include the following: lipedema almost exclusively appears in women, is symmetric, and spares the feet, whereas lymphedema is usually asymmetric, with swelling that may involve the entire limb, including the feet. Lipedema changes minimally with elevation or compression and is associated with pain and easy bruising. Obesity is characterized by increased fat distribution throughout the body. Chronic venous insufficiency can cause swelling and pain; however, it is not usually symmetric and commonly presents with varicose veins.

A striking finding was the high prevalence of endocrine disorders (80% of cases), including type 1 diabetes, hypothyroidism, and metabolic syndrome. This observation aligns with emerging hypotheses about hormonal influences in lipedema pathogenesis [17,19]. The presence of these comorbidities suggests that absolute hormone levels may be less important than hormone ratios or tissue-specific sensitivity [20]. The HLA-DQ2/DQ8 positivity in case 2 raises intriguing questions about potential autoimmune components, warranting further investigation [21]. Recent research has demonstrated that lipedema patients have significantly higher prevalence of HLA-DQ2 (47.4%) and HLA-DQ8 (22.2%) compared to the general population, suggesting a potential link between gluten sensitivity and lipedema inflammation [21]. This finding is particularly relevant as it may explain the clinical improvement observed with gluten-free dietary interventions in our cases and opens new avenues for personalized treatment approaches based on genetic markers.

Three patients (60%) reported positive family histories with affected female relatives, supporting genetic predisposition with possible autosomal dominant inheritance and sex-influenced expression [22,23]. The manifestation in males despite typical female predominance may indicate additional genetic or environmental modifiers requiring investigation.

Diagnostic challenges in male lipedema are amplified by low clinical suspicion. Our experience demonstrates the value of multifrequency bioimpedance analysis in objectively documenting disproportionate fat distribution. The consistent finding of higher fat percentages in the lower limbs compared to overall body composition provides crucial diagnostic support when clinical features alone may be questioned.

Conservative management yielded remarkable short-term results, with a mean weight reduction of 7.0 kg and volume reduction of 2.5 L over approximately six weeks. These outcomes exceed typical obesity management results and suggest that targeted lipedema interventions may be particularly effective [24,25]. The successful use of GLP-1 agonists (semaglutide) aligns with recent reports of their efficacy in lipedema [26], though specific mechanisms require further study. The implementation of gluten-free dietary approaches in some cases may be particularly beneficial for patients with HLA-DQ2/DQ8 positivity, as recent evidence suggests a potential inflammatory link between gluten consumption and lipedema symptoms in genetically susceptible individuals [21]. The potential link between gluten ingestion and non-celiac autoimmune conditions has been increasingly recognized [27], supporting the rationale for dietary interventions in HLA-positive lipedema patients.

Intradermal lymphoscintigraphy may prove valuable in differentiating lipedema from lymphatic disorders and warrants inclusion in future prospective studies.

Our findings have important clinical implications. Healthcare providers should consider lipedema in males presenting with disproportionate lower body fat, especially those with positive family histories or endocrine comorbidities. Objective assessment tools like bioimpedance analysis can support diagnosis when clinical suspicion exists. HLA-DQ2/DQ8 testing may be considered in selected cases, particularly those with autoimmune comorbidities or who may benefit from dietary interventions.

Study limitations include small sample size, retrospective design, short follow-up period (mean, 6.2 weeks), absence of histopathological confirmation, and lack of systematic HLA typing in all patients. Single-center experience may limit generalizability. Long-term outcomes and treatment durability remain unknown.

Future research priorities include large-scale prevalence studies, investigation of hormonal profiles and genetic factors such as HLA typing, randomized controlled trials of interventions such as gluten-free dietary approaches for HLA-positive patients, and long-term outcome assessment. The development of male-specific diagnostic criteria and treatment protocols may be warranted as more cases are identified.

## Conclusions

This case series confirms that lipedema affects men with classical phenotypic features comparable to female presentations. All five patients demonstrated bilateral, symmetrical lower extremity fat accumulation with characteristic sparing of the feet. The high prevalence of endocrine comorbidities and positive family histories suggests complex pathophysiological mechanisms requiring further investigation.

Conservative management, including dietary interventions, exercise programs, and adjunctive medical therapy, achieved significant short-term improvements in all patients. Early recognition through clinical assessment and objective measures such as bioimpedance analysis enables timely intervention.

Recognition of male lipedema challenges traditional gender-based diagnostic paradigms and emphasizes the need for inclusive clinical approaches. Healthcare providers should maintain awareness that lipedema can occur in men, particularly those with disproportionate fat distribution, family histories, or endocrine disorders. The potential role of HLA typing and dietary interventions represents an emerging area requiring further investigation.

Multicenter prospective studies using standardized diagnostic criteria are urgently needed to determine true prevalence, elucidate underlying mechanisms such as potential HLA associations, and establish evidence-based management guidelines for male lipedema. Inclusion of male patients in lipedema research will foster a more comprehensive understanding and optimize care for all affected individuals.
